# Association of serum levels of lipid and its novel constituents with the different stages of esophageal carcinoma

**DOI:** 10.1186/1476-511X-8-48

**Published:** 2009-10-29

**Authors:** Yutao Diao, Hao Li, Huiqing Li, Yingzhi Zhou, Qing Ma, Yan Wang, Dong Li

**Affiliations:** 1 College of Public Health, Shandong University, Jinan, PR China; 2 Institute of Basic Medicine of Shandong Academy of Medical Sciences, Jinan, PR China; 3 Department of Hematology, Tumor Center of Qilu Hospital, Shandong University, Jinan, PR China; 4 College of Economics, Shandong University, Jinan, PR China; 5 People's Hospital, Feicheng County, Shandong Province, PR China

## Abstract

**Background:**

The aim of the study was to evaluate the association of immunoglobulin G type of autoantibodies to oxidized low-density lipoprotein (oxLDL-lgG) and oxLDL-lgM with the progression of esophageal squamous cell carcinoma (ESSC).

**Methods:**

Residents from Feicheng, China aged 40 to 69 years were screened for esophageal lesions in a screening program conducted during the period of January 2008 to December 2006. There were 33 controls with normal esophageal squamous epithelium cells, 37 patients with basal cell hyperplasia, 47 with esophageal squamous cell dysplasia, and 43 with ESCC. All the participants were diagnosed by biopsy and histopathological examination. Adiponectin, oxidized low-density lipoprotein (oxLDL), autoantibodies against oxLDL (oxLDL-ab), OxLDL-lgG, and OxLDL-lgM were determined by enzyme linked immunosorbent assay (ELISA). Total cholesterol, High-density lipoprotein (HDL), triglyceride, serum albumin, and blood pressure were co-estimated. Analysis of covariance for lipid levels was used to control the influence of covariates.

**Results:**

The level of oxLDL-lgM increased gradually along with esophageal carcinoma progression. The oxLDL-lgM levels in the ESCC group were the highest after possible covariates were controlled. Binary logistic regression showed that oxLDL-lgM had a positive correlation with the development of esophageal carcinoma, while oxLDL and oxLDL-ab had a negative correlation with ESSC. No significant association between the levels of oxLDL-lgG and adiponectin and the different stages of ESSC was observed.

**Conclusion:**

The present study shows that the decreased oxLDL and oxLDL-ab and the elevated oxLDL-lgM serum levels may relate to the development and progression of ESSC.

## Background

Conflicting data have been reported on the association between low serum cholesterol levels and the risk for cancer mortality. Some observational studies show that low serum cholesterol is associated with an increased risk of cancer mortality [1-4], but some failed to establish a connection [5-8].

A meta-analysis of 18 cohort studies showed a significantly increased risk of total cancer death in men (but not in women) with total cholesterol concentrations less than 160 mg/dL(4.15 mmol/L) compared with those with levels at 160-199 mg/dL (4.15-5.16 mmol/L) [9].

There may be a causal link between low cholesterol and cancer. But it is possible that the associations between low blood cholesterol concentrations and risks of cancer are the consequences of confounding factors [3,4]. In addition, it is also possible that the associations follow a cause-and-effect relationship, possibly the preclinical cancer effect on cholesterol levels [10-12]. For instance, mortality from liver and colon cancer is significantly associated with a very low cholesterol level without any evidence of a preclinical cholesterol-lowering effect [13].

Although the mechanism is unclear, this phenomenon indicates that blood lipid levels are related to the development and progression of tumors. However, expression levels of serum cholesterol may not be the unique representative of the lipid markers.

The significance of lipid and its novel constituents such as adiponectin, immunoglobulin G and M types of autoantibodies to oxidized low-density lipoprotein (oxLDL-lgG and oxLDL-lgM) has been previously established in relation to cardiovascular disease [14,15]. Oxidized LDL, as an autoantigen, plays a crucial role in atherogenic lesion formation [16,17]. Serum oxLDL as well as the autoantibodies against it (oxLDL-ab) may be considered as biomarkers of lipid peroxidation [18]. There are two antibody isotypes to oxLDL, namely, the IgG and IgM autoantibodies. Although still controversial, overall evidence supports the notion that IgG autoantibodies to OxLDL are associated with pro-atherogenic properties and IgM autoantibodies to OxLDL with anti-atherogenic properties [19-21].

Meanwhile, studies also showed that adiponectin, oxLDL-lgG, and oxLDL-lgM may play a role in various types of malignancies [15]. Petridou, et al. studied the relationship between adiponectin and childhood myeloblastic leukemia [22], while Wang, et al. proposed that oxLDL autoantibody might be related to esophageal cancer [23]. Furthermore, it has been found in animal models that a high level of lipid peroxidation is closely associated with carcinogenesis [24,25].

It can be assumed that lipid and its novel constituents(oxLDL-ab, oxLDL-lgG and oxLDL-lgM) may also be associated with different stages in carcinogenesis proceeding. Pathological and epidemiological studies suggest that the malignant transformation of human esophageal mucosa is a progressive process that starts from normal epithelium to basal cell hyperplasia, followed by dysplasia, or carcinoma in situ, and finally to invasive esophageal squamous cell carcinoma (ESCC). Dysplasia, out of the 4 progressive stages is considered as an important precancerous lesion of esophageal cancer [26-28].

In this context, we carried out a program for the screening of esophageal lesions by endoscopic staining with 1.2% iodine solution in Feicheng, China between January 2004 and December 2006. Through the program, we obtained samples of 4 groups respectively having normal epithelium, basal cell hyperplasia, dysplasia, or carcinoma in situ, and ESCC. The foundation of these samples pressed us to establish the relationship of the serum levels of lipid-related constituents to these different lesions and carcinoma of the esophagus, especially the levels of oxLDL-lgG or/and oxLDL-lgM to the different stages in the progression of ESSC.

## Methods

### Study subjects

Between January 2004 and December 2006, we screened all the residents aged 40 to 69 in Feicheng, China, who agreed to participate in the program. We excluded those with liver diseases, diabetes, or cardiovascular diseases including coronary heart disease, angina pectoris, myocardial infarction, cardiac arrhythmia, and heart failure diagnosed by general medical check up, electrocardiogram, and abdomen supersonic inspection. All participants took part in the endoscopic staining with 1.2% iodine solution. Biopsies were taken from a non-staining area of the mucosa, and the samples underwent two separate pathologic evaluations carried out by two pathologists. Based on the examinations, the subjects were divided into four groups: the control group with normal esophageal squamous epithelium cells, the basal cell hyperplasia group, the esophageal squamous cell dysplasia group, and the ESCC group. The subjects in this study were randomly selected from the four groups. There were 33 subjects with normal esophageal squamous epithelium cells in the control group; 37 patients had basal cell hyperplasia, 47 had esophageal squamous cell dysplasia, and 43 had ESCC. Informed consent was obtained from all the subjects. The study protocol was approved by the local ethics committee.

### Measurements

An epidemiologic survey of the characteristics of the subjects such as name, gender, age, and smoking and drinking habits was conducted using a questionnaire. The body weight and height of each subject were measured, and the body mass index (BMI) was calculated as weight in kilograms divided by height in square meters. Blood pressure was measured by mercury sphygmomanometer. Hypertension was defined as having systolic pressure (SBP) ≥ 140 mmHg and/or diastolic pressure (DBP) ≥ 90 mmHg, or those who were already undergoing medications for hypertension.

### Blood Sampling and Biochemical Analysis

Venous blood samples were taken in the morning after an overnight fast of at least 12 hr. Plasma and serum were separated according to the different requirements. The specimens were kept frozen at -40°C until assayed. High density lipoprotein (HDL), total cholesterol (TC), and triglyceride (TG) levels were determined by enzymatic techniques. Low density lipoprotein (LDL) was calculated by the Friedewald formula [LDL = TC - HDL - TG/5]. Serum albumin was determined by the bromocresol green method. All the assays were performed under the instructions supplied by the corresponding manufacturers.

### Determination of serum ADP, oxLDL, oxLDL-ab, oxLDL-lgG, and oxLDL-lgM

An enzyme-linked immunosorbent assay(ELISA) for ADP, oxLDL, oxLDL-ab, oxLDL-lgG, and oxLDL-lgM was performed with ADP, oxLDL, oxLDL-ab, oxLDL-lgG and oxLDL-lgM ELISA kits purchased from ADL (Adliteram Diagnostic Laboratories Inc., USA). According to the manufacturer's instructions, using coated microtitration strips 96 wells, plasma was diluted 1:1 and incubated at room temperature for 1 hr in plates precoated with ADP, oxLDL, oxLDL-ab, oxLDL-lgG, or oxLDL-lgM, respectively. After washing 3 times, the plates were incubated with horseradish peroxidase (HRP) at room temperature for 30 min. After the removal of unbound conjugates by washing 3 times, tetramethylbenzidine (TMB) was added to the wells as a chromogenic substrate and incubated at room temperature for 10 min in the dark. Color development was stopped with stopping solution, and the absorbency was measured at 450 nm within 30 min. ADP, oxLDL, oxLDL-ab, oxLDL-lgG, or oxLDL-lgM titers were calculated by constructing a standard curve using the standards included in the respective kits. The concentrations of ADP, oxLDL, oxLDL-ab, oxLDL-lgG, and oxLDL-lgM in the samples were quantified in biomedical units as defined by the manufacturer. The intra-assay and interassay reproducibility (indicated by coefficients of variation) of the assay were 6, 5, 7, 6, and 10 percent, respectively.

### Statistical analysis

All statistical analyses were conducted using SPSS 15.0. The baseline characteristics were presented for quantitative data as mean (SD). The comparison in the four groups was performed using the Student's t test. The qualitative data were tested by the Chi-square test. The correlation between variables was tested using the Pearson correlation test depending on the distribution of the data. A general linear model analysis was used to control the influence of covariates when comparing the concentrations of ADP, oxLDL, oxLDL-ab, oxLDL-lgG, and oxLDL-lgM among the four groups. Multinomial logistic regression was applied to analyze the influential factors. Probability was significant at a level of ≤ 0.05.

## Results

### Characteristics of subjects

The clinical and biochemical characteristics of the studied population are shown in Table 1. The variables of age, albumin, total cholesterol (TC), high-density lipoprotein (HDL), low-density lipoprotein(LDL), oxLDL, oxLDL-ab, oxLDL-lgG, and oxLDL-lgM were significantly different among the four groups. There were no significant differences in the other variables.

**Table 1:** Characteristics of the Subjects

**variables**	**Control group**	**Basal cell hyperplasia**	**Dysplasia group**	**ESSC group**	**F/*x*^2^**	***p***
Age, years	49 ± 8	53 ± 8	56 ± 7	58 ± 7	10.286	0.000*
Sex, M/F	18/15	24/13	28/19	28/15	1.155	0.764
Hypertension, Y/N	18/15	20/17	18/29	21/22	2.893	0.408
BMI, kg/m^2^	22.90 ± 2.72	22.93 ± 2.90	22.06 ± 2.60	21.73 ± 3.31	1.728	0.164
SBP, mmHg	132 ± 26	132 ± 19	131 ± 20	127 ± 17	0.549	0.649
DBP, mmHg	86 ± 14	85 ± 12	82 ± 12	78 ± 14	2.325	0.077
Smokers, Y/N	15/18	17/20	24/23	24/19	1.119	0.773
Drinkers, Y/N	15/18	24/13	24/23	23/20	2.890	0.409
Albumin, g/L	38 ± 3	44 ± 8	40 ± 6	38 ± 8	8.161	0.000*
TC, mmol/L	3.45 ± 1.09	3.63 ± 0.83	3.62 ± 0.80	2.54 ± 0.96	13.684	0.000*
TG, mmol/L	0.61 ± 0.43	0.68 ± 0.46	0.57 ± 0.40	0.50 ± 0.32	1.481	0.222
LDL, mmol/L	0.12 ± 0.33	0.08 ± 0.27	0.09 ± 0.28	0.23 ± 0.15	11.439	0.000*
HDL, mmol/L	0.77 ± 0.22	0.91 ± 0.22	0.85 ± 0.21	0.67 ± 0.24	8.667	0.000*
ADP, ng/L	10.27 ± 10.98	11.18 ± 13.10	14.33 ± 13.38	12.98 ± 11.79	0.850	0.469
oxLDL, ng/L	33.66 ± 11.54	27.40 ± 10.33	30.27 ± 11.83	23.94 ± 10.72	5.264	0.002*
oxLDL-ab, U/ml	38.33 ± 26.44	38.97 ± 21.38	32.52 ± 12.87	26.59 ± 7.88	4.210	0.007*
oxLDL-lgG, ng/ml	12.54 ± 4.67	18.14 ± 10.47	16.56 ± 8.42	10.23 ± 6.78	8.453	0.000*
oxLDL-lgM, ng/ml	20.56 ± 5.99	23.27 ± 6.17	24.63 ± 6.35	26.60 ± 5.88	6.402	0.000*

* *p *< 0.05; Abbreviations: ESSC, esophageal squamous cell carcinoma; MBI, body mass index; SBP, 0 systolic blood pressure; DBP, diastolic blood pressure; TC, total cholesterol; TG, triglyceride; HDL, High-density lipoprotein; LDL, Low-density lipoprotein; ADP, adiponectin; oxLDL, oxidized low-density lipoprotein; oxLDL-lgG, immunoglobulin G type of autoantibodies to oxidized low-density lipoprotein; oxLDL-lgM, immunoglobulin M type of autoantibodies to oxidized low-density lipoprotein.

### Comparison of serum levels of ADP, oxLDL, oxLDL-ab, oxLDL-lgG, and oxLDL-lgM among the four groups

**Figure 1:**
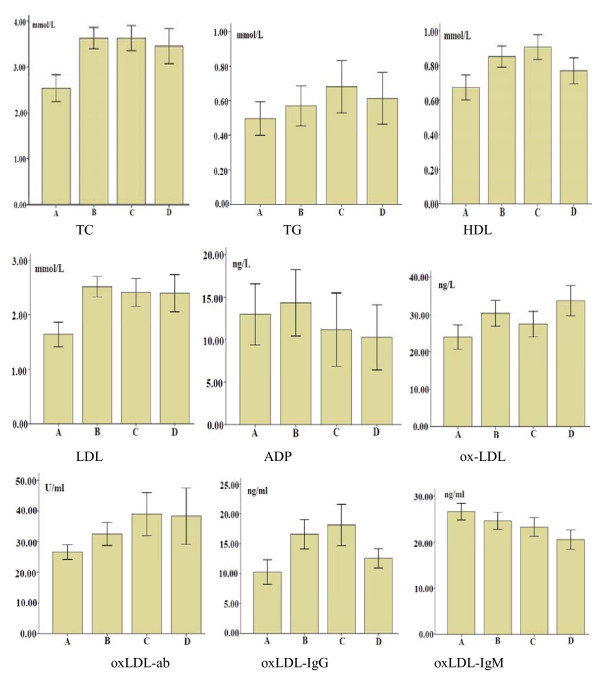
**Mean concentrations of serum biochemical indicators in group**. Mean concentrations of serum biochemical indicators in groups of ESSC (A), dysplasia (B), basal cell hyperplasia (C), and normal control (D). Adp, HDL, LDL, oxLDL, oxLDL-ab, oxLDL-IgG, oxLDL-IgM, TC, and TG respectively represent adiponectin, high-density lipoprotein, low-density lipoprotein, oxidized low-density lipoprotein, total antibodies to oxidized low density lipoprotein, oxidized low-density lipoprotein IgG, oxidized low-density lipoprotein IgM, total cholesterol, and total triglyceride.

A general linear model analysis showed that there were significant differences in the estimated marginal means of plasma oxLDL and oxLDL-lgM among the four groups. However, no significant differences were observed for ADP, oxLDL-ab and oxLDL-lgG after controlling the confounding variables i.e. age, TC, HDL, LDL, and albumin (Table 2). The results of the linear-by-linear association analysis showed that the blood serum levels of oxLDL and oxLDL-ab were decreased. However, the blood serum level of oxLDL-lgM increased with the histological spectrum of disease from normal to early invasive squamous cell carcinoma of the esophagus (Figure 1).

**Table 2:** Estimated Marginal Means and UNIANOVA Test of Apinectin, oxLDL, oxLDL-ab, oxLDL-lgG, and oxLDL-lgM in Four Groups^a^

	**ADP**	**oxLDL**	**oxLDL-ab**	**oxLDL-lgG**	**oxLDL-lgM**
ESSC group	11.699 ± 2.715	25.436 ± 1.951	28.123 ± 3.124	13.378 ± 1.214	26.519 ± 1.073
Dysplasia group	13.980 ± 1.867	30.130 ± 1.675	32.101 ± 2.681	15.645 ± 1.065	24.380 ± 0.921
Basal cell hyperplasia group	11.903 ± 2.202	26.629 ± 1.976	37.367 ± 3.164	15.553 ± 1.257	22.989 ± 1.087
Control group	11.614 ± 2.363	32.790 ± 2.120	38.742 ± 3.395	12.656 ± 1.349	21.329 ± 1.166
*F *test	0.332	2.739	2.098	1.445	3.503
*P*	0.802	0.045	0.103	0.232	0.017
Trend test^b^	1.518	9.421	10.412	2.789	17.137
*P*	0.218	0.002	0.001	0.095	0.000

Abbreviations: TC, total cholesterol; TG, triglyceride; ADP, adiponectin; HDL, High-density lipoprotein; LDL, Low-density lipoprotein; oxLDL, oxidized low-density lipoprotein; oxLDL-lgG, immunoglobulin G type of autoantibodies to oxidized low-density lipoprotein; oxLDL-lgM, immunoglobulin M type of autoantibodies to oxidized low-density lipoprotein.

^a ^Means (SE) for estimated marginal means; Covariates appearing in the model are evaluated at the following values: TG = 3.2961, LDL = 2.2317, HDL = 0.7992, Albumin = 40.2234, age = 54.55.

^b ^Linear --by --linear association test.

### Association of ADP, oxLDL, oxLDL-ab, oxLDL-lgG, and oxLDL-lgM with multistage development of esophageal carcinoma

As shown in Table 3, the Odds ratio (OR) of ADP, oxLDL, oxLDL-ab, oxLDL-lgG and oxLDL-lgM were associated with the different stages in the development of esophageal carcinoma (basal cell hyperplasia, dysplasia and early invasive cancer) after adjusted for age, albumin, TC, HDL and LDL.

**Table 3:** Multinomial Logistic Analysis for the Multistage Development of Esophageal Carcinoma

	**Unadjusted OR & 95%CL**			Adjusted OR & 95%CL*		
	
**variables**	**ESSC**	**dysplasia**	**Basal cell hyperplasia**	**ESSC**	**dysplasia**	**Basal cell hyperplasia**
ADP	1.02(0.98-1.06)	1.03(0.99-1.07)	1.01(0.97-1.05)	1.02(0.97-1.06)	1.03(0.99-1.07)	1.01(0.96-1.05)
oxLDL	0.92(0.88-0.94)	0.98(0.94-1.01)	0.96(0.92-1.00)	0.94(0.89-0.99)	0.97(0.93-1.01)	0.95(0.91-0.99)
oxLDL-ab	0.93(0.88-0.97)	0.98(0.96-1.01)	1.00(0.98-1.02)	0.96(0.91-1.00)	0.98(0.95-1.01)	1.00(0.98-1.02)
oxLDL-lgG	0.94(0.87-1.01)	1.07(1.01-1.14)	1.09(1.03-1.16)	1.03(0.94-1.13)	1.07(0.99-1.16)	1.07(0.99-1.15)
oxLDL-lgM	1.21(1.10-1.33)	1.15(1.05-1.27)	1.11(1.01-1.22)	1.18(1.06-1.31)	1.11(1.01-1.23)	1.08(0.98-1.20)

Abbreviations: TC, total cholesterol; TG, triglyceride; ADP, adiponectin; HDL, High-density lipoprotein; LDL, Low-density lipoprotein; oxLDL, oxidized low-density lipoprotein; oxLDL-lgG, immunoglobulin G type of autoantibodies to oxidized low-density lipoprotein; oxLDL-lgM, immunoglobulin M type of autoantibodies to oxidized low-density lipoprotein.

*:adjusted for age, albumin, TC, HDL, and LDL.

It is also indicated that the ORs for oxLDL-lgM were positively linked with the ESSC and dysplasia group. However, ORs for oxLDL and oxLDL-ab were negatively associated with the two groups, even if the ORs did not reached statistics significantly levels in dysplasia group.

## Discussion

In the present study, there was a significantly lower serum level of TC and HDL-c. Moreover, there were significantly higher serum levels of LDL-c in the ESSC group than those in the control group, which is similar to those previously found in the literature [1-5,10].

Plasma adiponectin levels were found to be lower in patients with many cancers, especially in upper gastric cancers, compared with those in the normal controls. It is inversely correlated with tumor size, depth of invasion, and tumor TNM stage, suggesting a potential role of adiponectin in the progression of gastric cancer [29]. There was no similar information for esophageal carcinoma. In the present study, we did not find plasma adiponectin levels to be related to the multistage development of esophageal cancer.

The predominant isotype of oxLDL antibodies isolated either from serum (free antibodies) or from precipitated soluble immune complexes (antigen-associated antibodies) is IgG, which is of the subclasses 1 and 3 [30,31]. A positive correlation between the levels of oxLDL-lgG antibodies and the different endpoints considered as evidence of atherosclerotic vascular disease has been reported by many studies [32,33]. In addition, the protective role of IgM oxLDL antibodies has been proposed in human cardiovascular diseases [25,34]. However, other studies have failed to support these findings [35-37].

Recent research works show that the antibody against oxLDL serum levels in patients with hepatocellular carcinoma [38] and squamous cell carcinoma of the esophagus [17] is lower than that in the normal controls. However, the isotypes of oxLDL-lgG and oxLDL-lgM were not separately detected in these reports.

The main finding of the present study is the descending trends of plasma oxLDL, oxLDL-ab, and oxLDL-lgG levels and the elevated trends of plasma oxLDL-lgM levels along with the series of histological spectrum of disease from normal to early invasive squamous cell carcinoma of the esophagus.

In recent years, there has been a growing body of evidence that states the excessive lipid peroxidation products including oxLDL and anti-oxLDL autoantibodies, which are reflected in the indicators of oxidative stress in vivo, may play a key role in cancer development [39,40]. Lipid peroxidation metabolites damage DNA and can seriously inhibit DNA repair capacity through their direct interaction with repair proteins [41]. Furthermore, they influence the intracellular redox equilibrium, affecting several crucial signal transduction pathways [42]. The oxLDL, shown either in vivo or in vitro, has a cytotoxic effect against endothelial cells and smooth muscle cells of the arterial wall, as well as a chemotactic activity for circulating monocytes [21]. Low serum levels of the oxLDL antibody can lead to uncontrolled cell proliferation and to the reduction of apoptosis, contributing to the induction of carcinogenesis. oxLDL increases the activity of C protein kinase (PKC), which is a serin-treonin kinase that acts on the cytoplasmatic transduction of the mytogenic signal, induced from GF [43]. The PKC exerts its action supporting the activity of P21 *ras *protein involved in the pathogenesis of many humans tumors [44]. oxLDL is also able to induce the production and delivery of other growth factors (GF) such as that derived from platelets (PDGF), probably through interleukin 1 and TNF-β. It has been demonstrated that PDGF, codified from c-sys oncogenes, seems to be involved in the uncontrolled growth of many tumors [45]. The anti-oxLDL autoantibodies may be activated through all these different ways to induce the development and progression of esophageal cancer.

In this study, all subjects were diagnosed by histopathology, which ruled out the possibility of the misclassification error. In the experimental determination of the indicators, we used less than 10 percent of the coefficient of variation as quality control. In order to avoid the occurrence of collinearity, a covariance analysis was used in the analysis. The variance inflation factor(VIF) of ADP, oxLDL, oxLDL-ab, oxLDL-lgG, and oxLDL-lgM were 0.232, 0.880, 0.910, 0.867, and 0.984, respectively. Therefore, the main results of this study were neither biased nor by chance.

In the present study, we found that the descending levels of oxLDL and oxLDL-ab may negatively related to the different stages of the development of esophageal cancer while the elevated levels of oxLDL-lgM has a positive potence, which is contrary in the case of their relations to cardiovascular and cerebrovascular diseases revealed in previous literature.

However, the isotype antibodies against oxLDL serum levels in cancer cannot be defined in universal terms. Furthermore, large clinical sample studies are required in order to elucidate whether oxLDL as well as oxLDL antibodies play a causative or merely consequential role in the cancer process, and to designate a novel strategy for cancer prevention and therapy.

## Conclusion

This study shows that the decreasing oxLDL and oxLDL-ab negatively relate to the different stages of the development of esophageal squamous cell carcinoma while the elevated oxLDL-lgM serum levels may positively relate to the development and progression of ESSC. This conclusion may be helpful for cancer prevention and therapy.

## Abbreviations

TC: total cholesterol; TG: triglyceride; ADP: adiponectin; HDL: High-density lipoprotein; LDL: Low-density lipoprotein; oxLDL: oxidized low-density lipoprotein; oxLDL-lgG: immunoglobulin G type of autoantibodies to oxidized low-density lipoprotein; oxLDL-lgM: immunoglobulin M type of autoantibodies to oxidized low-density lipoprotein; ESCC: esophageal squamous cell carcinoma; oxLDL-ab: antibodies to oxidized low density lipoprotein

## Competing interests

The authors declare that they have no competing interests.

## Authors' contributions

Members listed below made their respective contributions to this manuscript.

Professor HQL designed the skeleton of this study, supervised the epidemiologic survey of the characteristics of the subjects, performed the statistical analysis and drafted the manuscript. HL, YTD, YZZ, QM and YW carried out the immunoassays in addition to biochemical analysis, whereas HL and YTD also participated in the compiling of this manuscript. DL engaged in the screening of esophageal lesions, inspected the subject(including some inpatients)enrolled in this study. All authors read and approved the final manuscript.
